# Selective Overview of 3D Heterogeneity in CMOS

**DOI:** 10.3390/nano12142340

**Published:** 2022-07-08

**Authors:** Cheng Li, Zijin Pan, Xunyu Li, Weiquan Hao, Runyu Miao, Albert Wang

**Affiliations:** Department of Electrical and Computer Engineering, University of California, Riverside, CA 92521, USA; cli043@ucr.edu (C.L.); zpan015@ucr.edu (Z.P.); xli400@ucr.edu (X.L.); whao008@ucr.edu (W.H.); rmiao008@ucr.edu (R.M.)

**Keywords:** flying noise, isolation, metal wall, ESD protection, graphene, gNEMS, interconnects, magnetic core, inductor, nano-crossbar array, heterogeneous integration, heterogeneity

## Abstract

As the demands for improved performance of integrated circuit (IC) chips continue to increase, while technology scaling driven by Moore’s law is becoming extremely challenging, if not impractical or impossible, heterogeneous integration (HI) emerges as an attractive pathway to further enhance performance of Si-based complementary metal-oxide-semiconductor (CMOS) chips. The underlying basis for using HI technologies and structures is that IC performance goes well beyond classic logic functions; rather, functionalities and complexity of smart chips span across the full information chain, including signal sensing, conditioning, processing, storage, computing, communication, control, and actuation, which are required to facilitate comprehensive human–world interactions. Therefore, HI technologies can bring in more function diversifications to make system chips smarter within acceptable design constraints, including costs. Over the past two decades or so, a large number of HI technologies have been explored to increase heterogeneities in materials, technologies, devices, circuits, and system architectures, making it practically impossible to provide one single comprehensive review of everything in the field in one paper. This article chooses to offer a topical overview of selected HI structures that have been validated in CMOS platforms, including a stacked-via vertical magnetic-cored inductor structure in CMOSs, a metal wall structure in the back end of line (BEOL) of CMOSs to suppress global flying noises, an above-IC graphene nano-electromechanical system (NEMS) switch and nano-crossbar array electrostatic discharge (ESD) protection structure, and graphene ESD interconnects.

## 1. Introduction

There is no question that semiconductors have reshaped human society. Revolution in microelectronics has transformed the modern world into the information technology (IT) era and, most recently, advances in semiconductors are rapidly transitioning our lives into the internet-of-everything (IoET) age. All these changes were essentially triggered by inventions of the Ge transistor in 1947 [1,2,3], followed by integrated circuits (ICs) in Ge and Si around 1958 and 1959 [4,5]. Most importantly, it was the birth of Si complementary metal-oxide-semiconductor (CMOS) IC technology in 1963 [6], due to its scaling-enabled integration and economic advantages, that drove the IT revolution into the fast lane. Unfortunately, the scaling-based continuous advances in CMOS IC technologies, mostly driven by Moore’s law [7], seem to be slowing down. On the other hand, demands for higher performance (e.g., speed) and more complexity (e.g., functions) of chips, mainly driven by data-centric IoET systems and applications, have been continuously increasing. It is generally agreed upon that heterogeneous integration is an emerging technology that offers a viable solution, alternative to classic scaling of various kinds, to continuously enhance performance of Si-based CMOS chips in beyond-Moore time [8]. The reason is that the performance of advanced chips goes well beyond classic logic functions. Instead, future chips require more functionality and higher complexity to facilitate the whole information chain, spanning from signal sensing, conditioning, and processing, to data storage and computing, to communications, control, and actuation to support human-in-the-loop cyber-physical systems (HCPS) and applications empowered by smart chips. In principle, heterogeneous integration is a technology to substantially enhance function diversifications and performance specifications, in aggregation, by heterogeneously integrating components, separately manufactured in different materials systems for individual optimization, into a higher-level assembly, i.e., at wafer scale relevant to IC chips, to accommodate the increasing demands for higher performance and more complexity of smart system chips. Towards this end, great efforts have been devoted to developing various heterogeneous integration (HI) technologies and structures to bring both characteristic boosters and non-IC functionalities into the mature, dominant Si CMOS platform. For example, new performance boosters may be novel materials in a metal-oxide-semiconductor field-effect transistor (MOSFET) channel to increase carrier mobility or a gate-all-around nanowire transistor to improve drive per footprint, while sensors, micro-electromechanical system (MEMS), and photonics can bring more functionality to Si CMOSs, and also bio-inspired devices may change the computing paradigm [9,10]. Over the past two decades, numerous HI technologies have been explored to increase heterogeneity in materials, technologies, devices, circuits, and system architectures, making it practically impossible to provide one single comprehensive review of the field in one paper. This article provides a topical overview of selected HI structures developed in CMOS platforms to boost CMOS chip performance, which include a stacked-via vertical magnetic-cored inductor structure in radio-frequency (RF) CMOS (Section 2), a metal wall structure in CMOS back end of line (BEOL) for global flying crosstalk isolation (Section 3), an above-IC graphene NEMS switch for electrostatic discharge (ESD) protection (Section 4), a phase-changing nano-crossbar array ESD protection structure (Section 5), and graphene nanoribbons for ESD interconnects (Section 5). This overview means to showcase both potentials and feasibility of emerging HI technologies and structures to make CMOS chips smarter without exceeding design, fabrication, energy, or economic constraints.

## 2. Stacked-Via Vertical Magnetic-Cored Inductor

The proliferation of wireless communications was enabled by radio-frequency (RF) ICs, which benefited critically from RF CMOS technology. On one hand, aggressive scaling of CMOS technologies led to higher frequencies (i.e., *f*_T_, *f*_max_) in Si CMOSs while retaining its most important feature, i.e., high integration, hence low costs. It was RF CMOSs that made high-performance RF ICs widely available to deliver affordable wireless gadgets, e.g., smartphones and wireless routers. On the other hand, not every device in RF ICs can be scaled down following Moore’s law. Inductive devices, e.g., inductors, which are practically indispensable to RF ICs, cannot be scaled down aggressively like MOSFETs. The main barrier is associated with signal energy losses. Shrinking the metal spiral of an IC inductor means a narrower metal wire, which leads to higher series resistance that results in more electrical energy loss, and thus poorer Q-factor. A poor Q-factor is very disadvantageous to many RF ICs. In addition, typical on-chip inductors are fairly large. Therefore, RF system-on-a-chip (SoC) has been impractical (technically and economically) in RF IC designs. Substantial research gone into addressing this RF IC design challenge. For example, substrate engineering and MEMS structures have been used to reduce substrate losses and improve Q-factor, e.g., using a suspended spiral over a deep cavity in the substrate [11]. Alternatively, magnetic media have been introduced into an inductor structure aiming to enhance magnetic flux and minimize magnetic energy loss, hence avoiding significant degradation in Q-factor, e.g., using planar solenoids or lateral magnetic films [12]. Generally, these special inductors are large and have complicated device structures.

**Figure 1:**
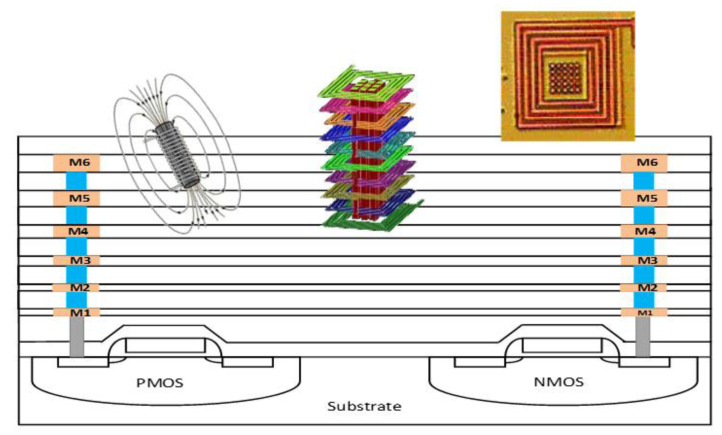
Concept of a stacked-via vertical magnetic-cored inductor in CMOS back end. Insets: a solenoid, simulated multiple-layer spiral inductor with vertical magnetic bar array and a prototype in a CMOS.

**Figure 2:**
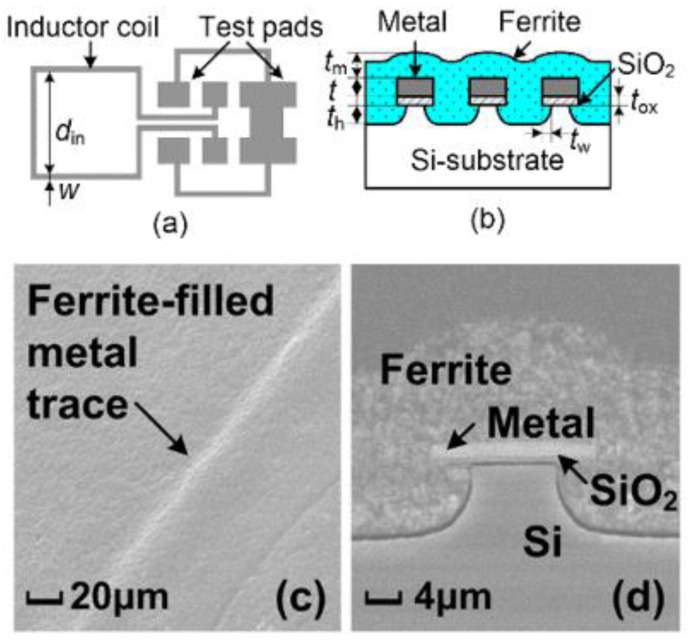
Prototype ferrite-partially-filled inductor fabricated: (**a**) device with GSG pads, (**b**) X-section, (**c**) Ni-Zn-Cu ferrite coating coil trace by SEM, and (**d**) X-section of Ni-Zn-Cu partially-filled metal trace by SEM [16].

**Figure 3:**
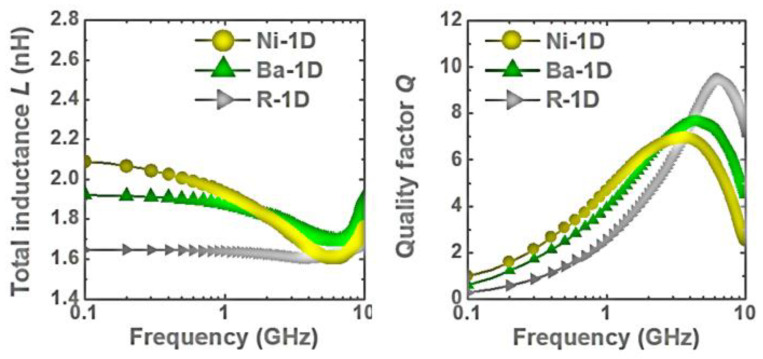
Measured L and Q for ferrite-partially-filled inductor prototypes: (**Left**) total L shows significant improvement for Ni-Zn-Cu sample at 0.1–5 GHz and Co_2_Z-type sample at 0.1–10 GHz over the air-cored references, and (**Right**) Q-factor shows significant improvements for Ni-Zn-Cu device at 0.1–3.5 GHz and Co_2_Z-type device at 0.1–4 GHz over the reference [16].

**Figure 4:**
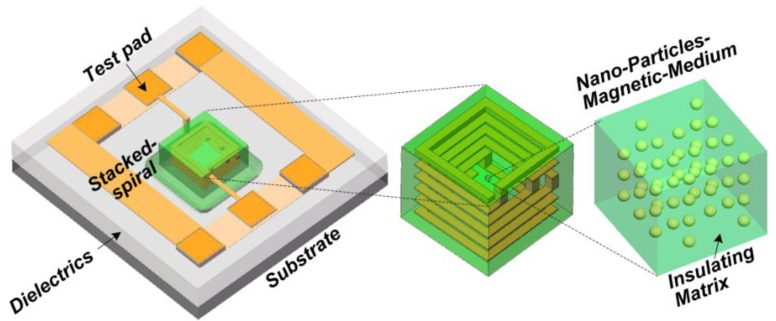
A 6-Al-layer stacked spiral inductor filled with nanomagnetic particles in a CMOS [22].

**Figure 5:**
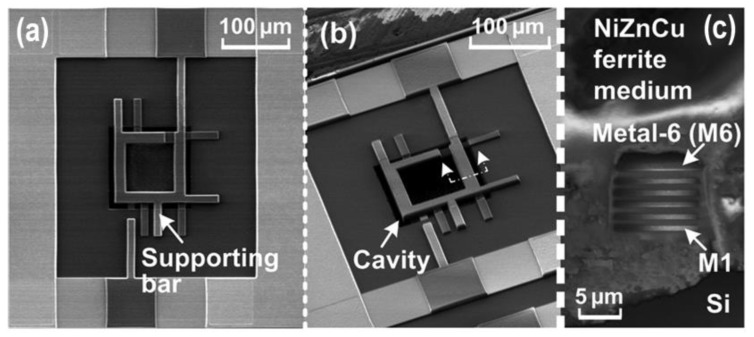
SEM photos for a vertical nanomagnetic particle-filled 6-Al-layer stacked spiral inductor fabricated in a 180 nm CMOS: (**a**) top view, (**b**) tilt view, and (**c**) cross-section view [20].

**Figure 6:**
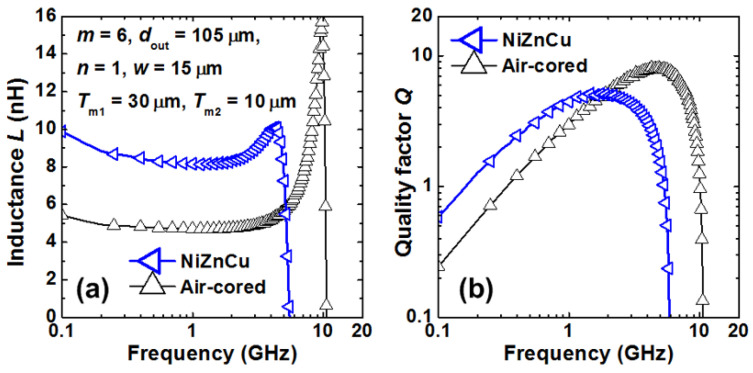
Measured performance improvement of 6-Al-layer (m = 6) single-turn (*n* = 1) solenoid-shaped stacked-spiral vnM-L inductors over the air-cored references: (**a**) L, (**b**) Q-factor. (d_out_: coil out diameter; w: line width) [20].

**Figure 7:**
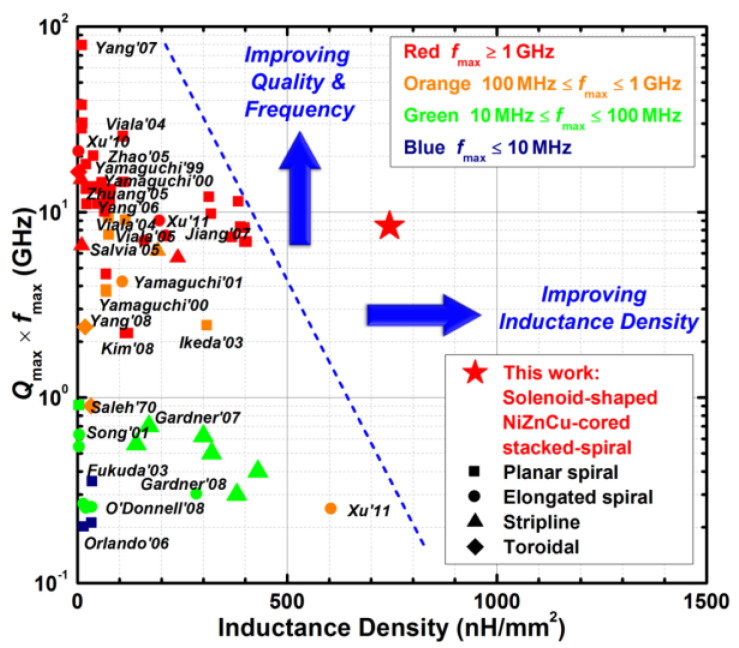
*Q*_max_ X *f*_max_ *versus* *L*-density chart for the vnM-L inductor and the published state-of-the-art results of lateral magnetic inductors, as well as several high-*Q* air-cored inductors [22].

**Figure 8:**
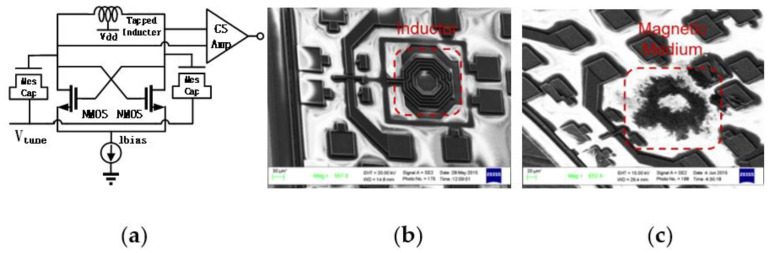
An LC-VCO in 180 nm SOI CMOS using magnetic-cored inductor: (**a**) schematic, (**b**) without magnetic core, and (**c**) with magnetic core for the inductor [24].

**Figure 9:**
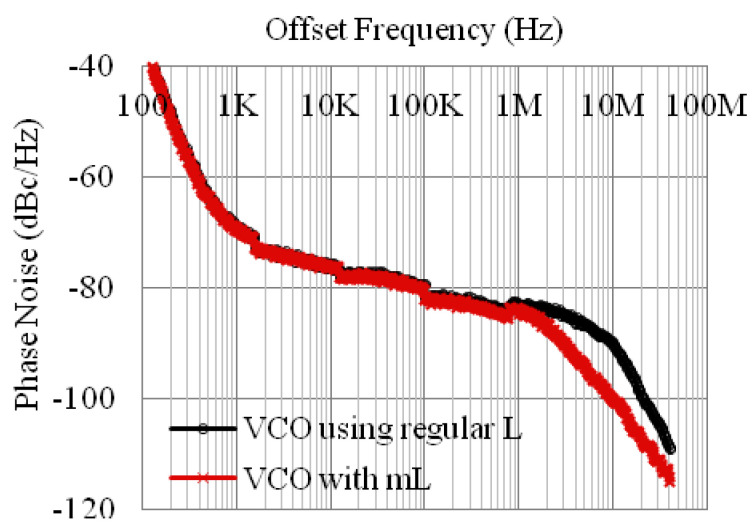
Measured phase noises for the LC-VCO IC samples with V_tune_ = 2.25 V [24].

One big question that an RF IC designer may ask is whether is it possible to design transistor-sized on-chip inductors with moderate inductance (L) and good Q-factor operating at multi-GHz frequencies to practically realize RF SoC in CMOSs. A new on-chip spiral inductor structure with a vertical stacked-via magnetic core bar array was invented and demonstrated in CMOSs to answer this RF IC call, as depicted in Figure 1 [13]. This vertically magnetic-cored inductor structure emerged from a hypothesis that an ideal discrete solenoid inductor would be shrunk to a needle of transistor dimensions, which could then be poked into the BEOL deck in a CMOS with multilayer metal (e.g., Cu) interconnects, and a transistor-sized vertical spiral inductor with a magnetic core would then be formed, being a miniaturized mimic of an ideal discrete solenoid. Using a CMOS process flow, a magnetic core bar array can be formed readily by replacing vias within an inductor by the desired magnetic materials layer by layer, hence creating a stacked-via vertical magnetic core bar array for better magnetic flux control while utilizing mature CMOS back-end processes [13]. The new vertical magnetic-cored inductor concept was validated experimentally in several steps [14,15,16,17,18,19,20,21,22,23,24]. First, research was conducted to explore different magnetic materials and their synthesis techniques to understand how materials’ compositions and processing methods would affect the magnetic characteristics, including complex permeability (μ = μ′ + jμ″) and frequency behavior, which generally affect ferromagnetic resonance frequency (*f*_FMR_ at μ″_max_), L, Q, *f*_max_ (the operating frequency at Q = Q_max_), and self-resonance frequency (*f*_0_). In general, magnetic materials of higher μ′ and lower μ″ extended to higher frequency ranges are preferred for on-chip inductors with higher inductance density (L-density) and Q, and are able to operate at a higher frequency (multi-GHz and beyond). This was confirmed by simulation and in experiments [14,18,19]. In the experiments [18,19], various ferrite materials were synthesized and studied using spiral inductors, as shown in Figure 2. The ferrite compositions used in the prototypes included Ni-Zn-Cu, YIG (Y-Fe-O), and Co_2_Z families with high *f*_FMR_ and compositions fine-tuned. The inductors were ferrite-partially-filled (Figure 2b) fabricated in a low-temperature CMOS process flow (Figure 2c,d). Figure 3 depicts the measured L and Q characteristics in the frequency domain for prototype inductor devices showing substantial improvements in both L and Q to high frequencies for inductors integrated with Ni-Zn-Cu (Ni_0.3_Zn_0.6_Cu_0.1_Fe_2_O_4_) at 0.1–5 GHz (up to +35% in L and +250% in Q) and Co_2_Z-type (Ba_3_Co_2_Fe_24_O_41_) at 0.1–10 GHz (up to +22% in L and +149% in Q) over the air-cored reference device. YIG samples showed good improvement in L, though significant degradation in Q, due to its high μ″. Second, the new vertical magnetic-cored inductor concept was then validated using stacked spiral inductors designed in a foundry 180 nm 6-Al metal CMOS [20,21,22]. Figure 4 depicts the schematic for the 6-Al-layer spiral inductor with a vertical nanomagnetic particle-filled core (nvM-L), with its fabricated device photo shown in Figure 5. To achieve high μ_eff_′, low μ_eff_″ and high *f*_FMR_, NiZnCu (Ni_0.25_Cu_0.25_Zn_0.5_Fe_2_O_4_) ferrite synthesized as nanoparticles (diameter, *d*~350 nm) were used as the vertical magnetic core. Figure 6 depicts the measured L and Q in the frequency domain for prototype devices, showing significant increase in L (more than +70%) to 5.2 GHz and improvement in Q to 1.6 GHz, respectively. The expected improvement in Q was much higher than that observed per simulation, and the lower Q increase in measurement was mainly attributed to the damage to the Al spirals during post-CMOS dry etching to create a deep hole inside the inductor coils of six metal layers in the university lab. The results showed that, with the vertical nanoferrite core, L-density of ~825 nH/mm^2^ was achieved, which means that a vnM-L device can be substantially shrunk in designs, e.g., ~80% size reduction of L~9nH. If more advanced CMOS technologies are used, more Cu metal layers can make a better and smaller vertical magnetic-cored inductor. Figure 7 shows that the nvM-L compares favorably to the then-state-of-the-art magnetic-enhanced inductors in terms of the figure of merit of *Q*_max_ X *f*_max_ *versus* *L*-density. Third, the magnetic-cored inductor concept was further validated in voltage-controlled oscillator (VCO) ICs [23,24]. Figure 8 depicts a 2.22–2.92 GHz LC-VCO designed and fabricated in a foundry 180 nm silicon-on-insulator (SOI) CMOS with a magnetic-cored inductor made by post-CMOS processes. The single-spiral magnetic-cored inductor features a higher L-density of ~17%, making it much smaller than a normal inductor of the same inductance. Measurement confirmed full circuit functions for the VCO using a magnetic-core inductor, including good phase noise, as shown in Figure 9. It is noteworthy that though the prototype of the vertical magnetic-cored inductor still needs improvement, it readily shows the potential to make high-quality transistor-sized on-chip inductors to enable large single-chip RF SoCs through heterogeneous integration of magnetic media into a Si CMOS platform. Two main challenges are to be addressed as future directions: first, novel nanomagnetic material synthesis to achieve higher μ′ and lower μ″ to beyond 10 GHz operations; second, process techniques to prevent any contamination in CMOS manufacturing.

## 3. In-BEOL Metal Wall Flying Crosstalk Isolation Structure

It is well known that crosstalk (a.k.a. noise coupling) through a conductive substrate (i.e., in-substrate noises) is a major problem to noise-sensitive analog and RF ICs. Various mature design techniques have been developed and adopted to isolate in-substrate global crosstalk in mixed-signal and RF ICs, e.g., double guard rings, deep trenches, buried ground plane, MEMS structures, substrate backside cavity, and high-resistivity substrates, etc. [25,26,27,28,29,30,31,32]. However, while these in-substrate noise-isolation techniques are very efficient in blocking in-substrate interference, they cannot suppress the global crosstalk in the BEOL deck through the massive and complex metal interconnects (called flying noises), which were believed dominant, accounting for up to ~80%, in the whole-chip noise coupling, particularly for complex chips at advanced technology nodes [33].

**Figure 10:**
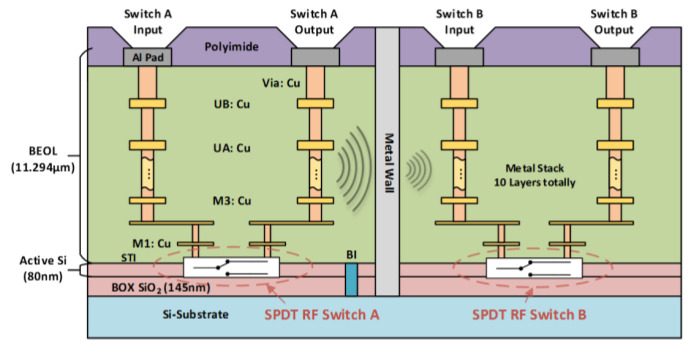
Cross-section view of the in-BEOL metal wall flying crosstalk isolation structure illustrated in SOI CMOS [35].

**Figure 11:**
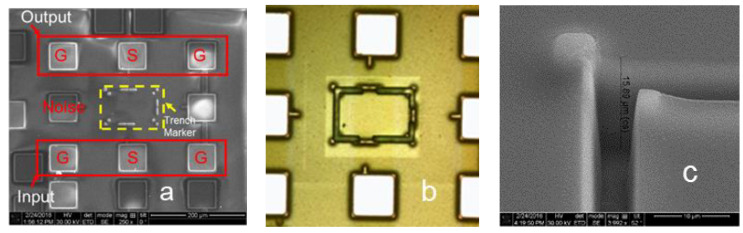
SEM photos for the in-BEOL metal wall structure in an amplifier IC designed in a 180 nm SOI CMOS: (**a**) MPW die showing the planned metal wall enclosure (marked in yellow dashed line), (**b**) the metal wall structure formed in post-CMOS fabrication, and (**c**) zoom in of the deep trench before metal filling [34].

**Figure 12:**
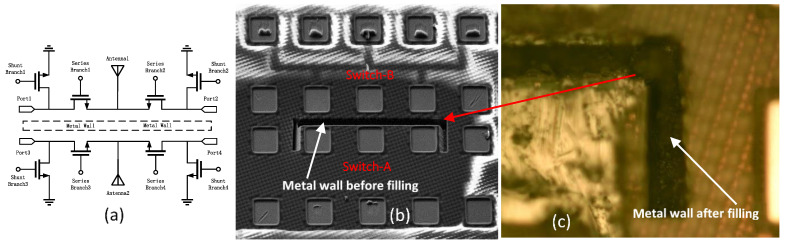
In-BEOL metal wall structure in an SPDT RF switch IC designed in a 45 nm SOI CMOS: (**a**) SPDT schematic, (**b**) partial metal wall trench etched by FIB, and (**c**) zoom in of the silver-filled metal wall structure [35].

**Figure 13:**
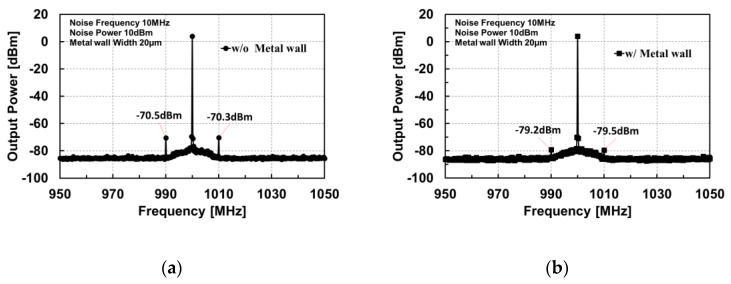
Measured output of the amplifier circuits: (**a**) without noise isolation, and (**b**) with metal wall isolation [34].

**Figure 14:**
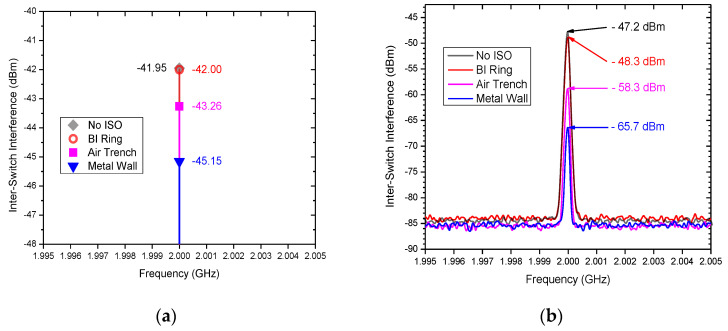
Crosstalk comparison of SPDT splits: (**a**) HFSS-ADS cosimulation, and (**b**) measurement (0 dBm input to Switch A; interference measured at output of Switch B) [35].

A unique in-BEOL metal wall crosstalk isolation technique was developed to effectively suppress global flying noises through metal interconnects in the back end [34,35]. Figure 10 depicts the concept of the novel in-BEOL metal wall noise-isolation structure, which is a deep trench circle (or partial) etched into the BEOL deck that is filled with metal to form an in-BEOL metal wall enclosure to isolate one circuit block from the other in a die. The concept structure was experimentally validated in two circuit designs, one amplifier IC designed in a foundry 180 nm FD-SOI CMOS [34] and the other being an SPDT RF switch IC fabricated in a foundry 45 nm SOI CMOS [35]. The in-BEOL metal wall structures were fabricated in post-CMOS processing after receiving the MPW dies from the foundry. Figure 11 shows the die photos for the amplifier IC case and Figure 12 gives the schematic and die photos for the SPDT circuit. In the prototypes, the deep trench was created using a focused ion beam (FIB, mill current of 30 kV/18 nA) that was then filled by silver nanopowder (99.99%, 80–100 nm) to form the metal wall enclosure. It was found that the property of the filling metal can be critical to electromagnetic isolation. The designs were guided by HFSS-ADS cosimulation. Figure 13 depicts the measured third-order intermodulation (IM3) for the first amplifier circuit, which shows a reduction of ~9 dBm in IM3 interference. In the second design of SPDT ICs, the post-CMOS process for making the metal wall structures was improved and an SPDT split using the foundry-recommended in-Si BI-ring (buried isolation) noise-isolation option was also included for comparison. Figure 14 shows the crosstalk characteristics at the output of Switch B (victim) due to the interference coming from Switch A (noise generator at 0 dBm input) by simulation and measurement. It is readily observed that the in-BEOL metal wall structure achieved a reduction in flying crosstalk of ~18.5 dB (i.e., ~98.6% noise suppression in linear scale). The prototypes confirmed that the in-BEOL metal wall crosstalk isolation structure is very efficient in blocking global flying noises on a chip.

## 4. Graphene NEMS ESD-Protection Structure

**Figure 15:**
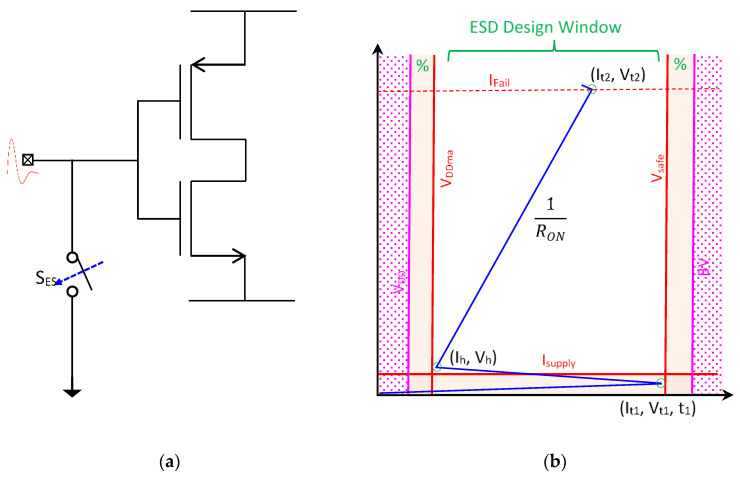
ESD-protection device concept and ESD design window: (**a**) ideal ESD-protection switch, and (**b**) ESD design window showing ESD-critical parameters.

**Figure 16:**
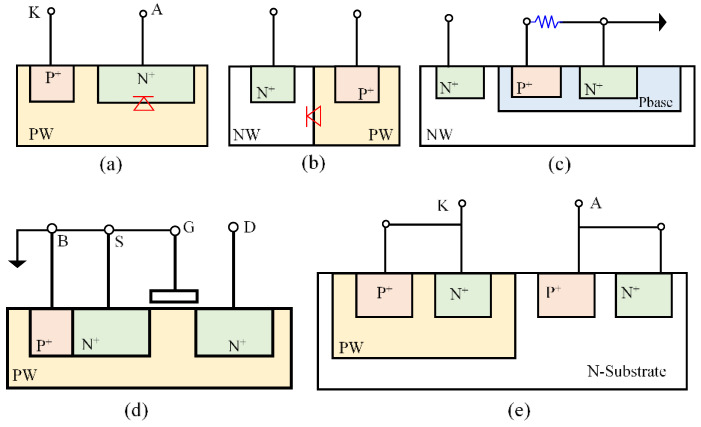
Traditional in-Si PN-based ESD-protection structures: (**a**) vertical diode, (**b**) lateral diode, (**c**) BJT, (**d**) MOSFET, and (**e**) SCR, as well as their derivatives.

Electrostatic discharge (ESD) failure is a major IC reliability problem that causes the industry billions of dollars of annual revenue loss. On-chip ESD protection is hence required for all ICs [36]. In principle, an ESD-protection device acts like a controlled switch that is connected to bonding pads on an IC die. As depicted in Figure 15, an ESD-protection device remains in an OFF state during normal IC operations so that it will not affect chip functions. During an ESD event, a fast and strong incident ESD transient appearing at an IC pad will trigger the ESD-protection device (i.e., ON), creating a low-resistance conduction path to discharge the ESD pulse to protect the IC. In ESD-protection designs, the ESD-critical parameters, including triggering voltage, current, and time (V_t1_, I_t1_, t_1_), holding voltage and current (V_h_, I_h_), discharging resistance (R_ON_), and thermal breakdown voltage and current (V_t2_, I_t2_), must be carefully designed to comply with the ESD design window in order to provide adequate on-chip ESD protection [37,38,39,40]. Unfortunately, ESD design overhead always exists, which includes ESD-induced parasitic capacitance (C_ESD_), leakage (I_leak_), noises and noise coupling, as well as Si area consumption and physical design difficulty in layout associated with large ESD devices [39,40,41,42]. The ESD design overhead problem is rapidly becoming unacceptable to large and complex ICs implemented at advanced technology nodes [39]. It is understood that the root cause of the ESD design overhead is the traditional in-Si PN-junction-based ESD-protection structure, as depicted in Figure 16, which has been commonly used for about 60 years. To fundamentally address the ESD design overhead challenge, revolutionary ESD-protection mechanisms and device structures are needed, towards which several novel non-PN-based ESD protection concepts have been reported.

**Figure 17:**
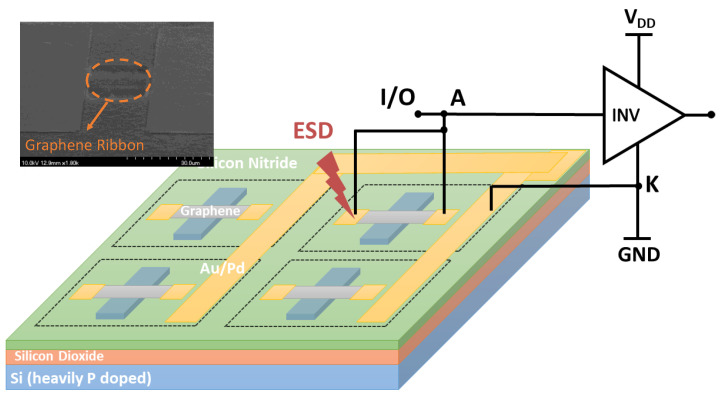
A new gNEMS ESD switch array structure for on-chip ESD protection (Inset: SEM photo for a prototype device fabricated).

**Figure 18:**
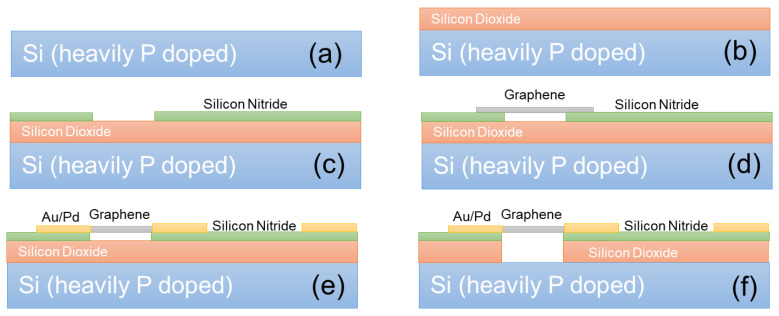
A CMOS-compatible process for fabricating gNEMS devices: (**a**) preparation of doped silicon, (**b**) thermal oxidation of silicon, (**c**) open a window in Si_3_N_4_, (**d**) transfer and pattern the graphene ribbon, (**e**) deposit the metal pad, and (**f**) HF etching SiO_2_ to release the graphene membrane to form the gNEMS device.

**Figure 19:**
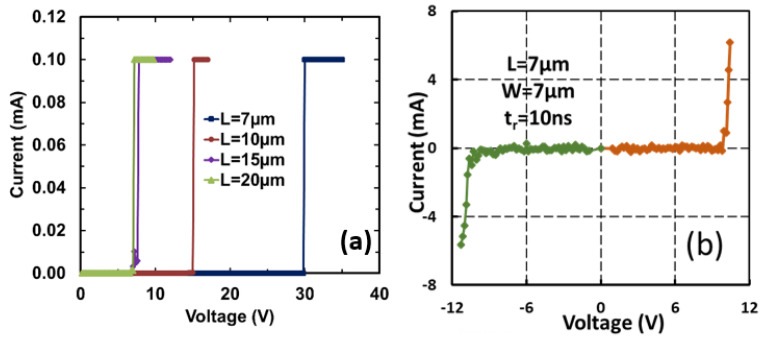
Measured conduction characteristics for gNEMS prototypes: (**a**) DC sweeping test showing static switching effect with turn-on voltage affected by the graphene membrane length (7.0, 7.6, 15, 29.8 V), and (**b**) TLP test showing dual-directional transient ESD discharging I-V behavior [43].

**Figure 20:**
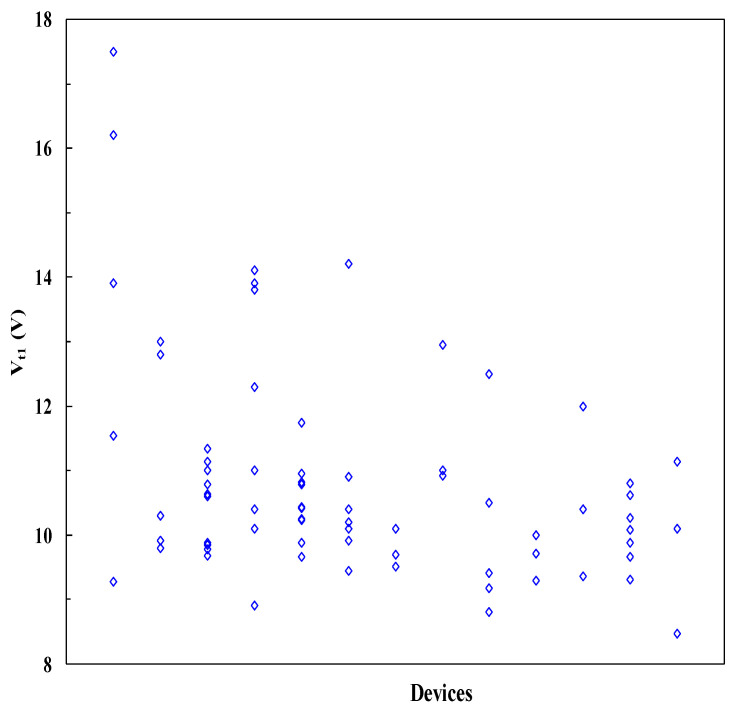
TLP measurement shows a wide range of ESD V_t1_ values for prototype gNEMS switches, adjustable by device design variations [36].

**Figure 21:**
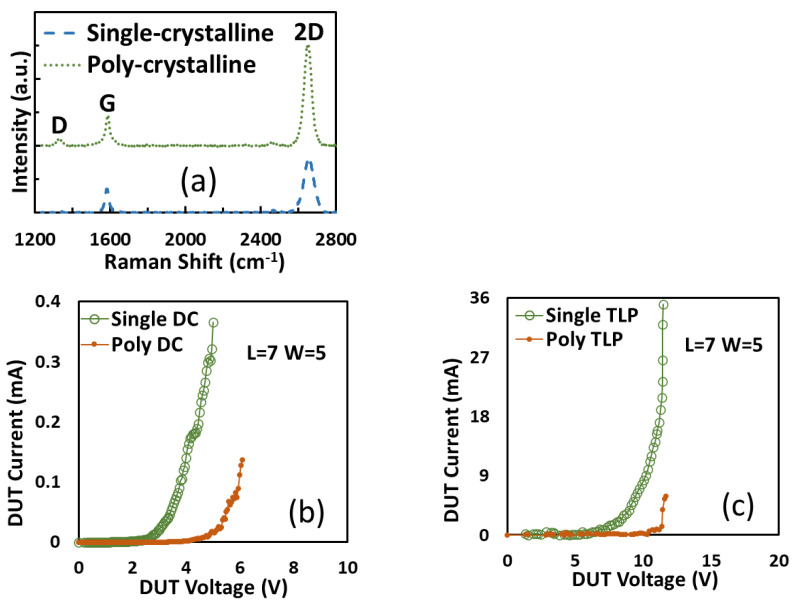
Measurement comparison of gNEMS devices made in single-crystal and polycrystal graphene films: (**a**) Raman spectrum, (**b**) DC sweeping test, and (**c**) TLP test [44].

**Figure 22:**
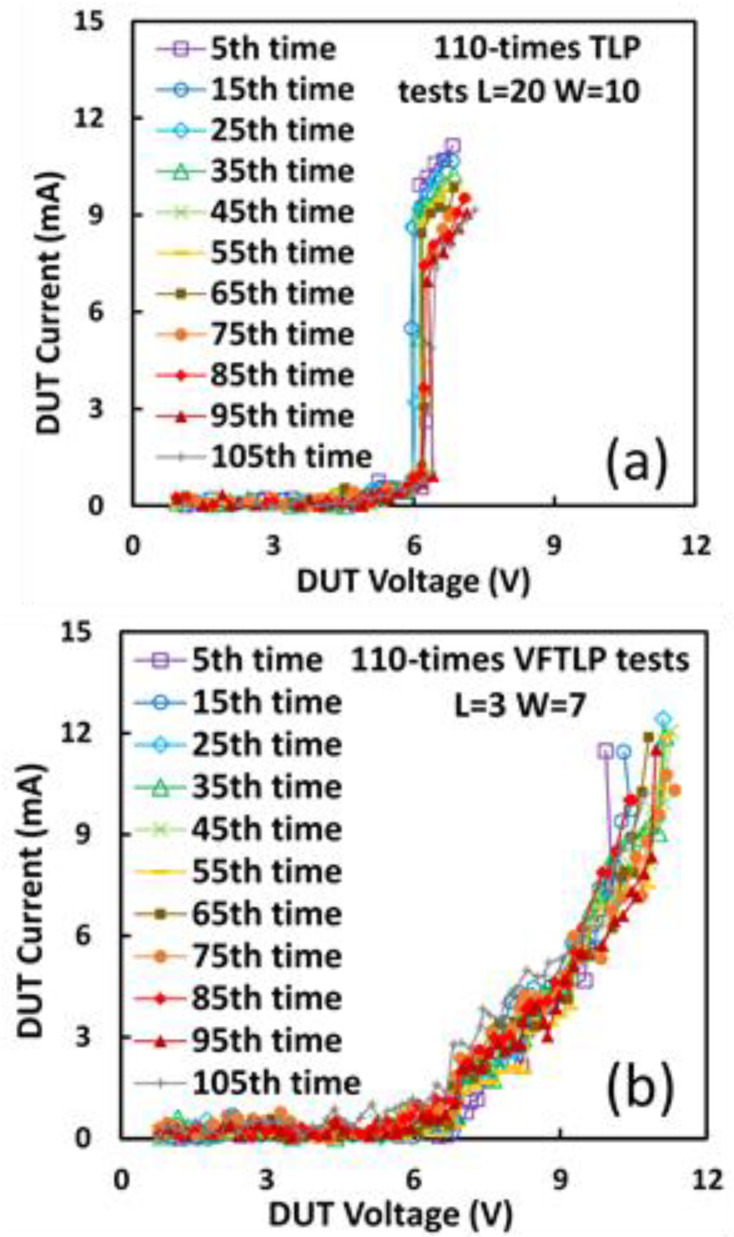
Durability evaluation of single-crystal graphene gNEMS devices by 110-fold ESD stress tests: (**a**) TLP zapping, and (**b**) VFTLP zapping [44].

**Figure 23:**
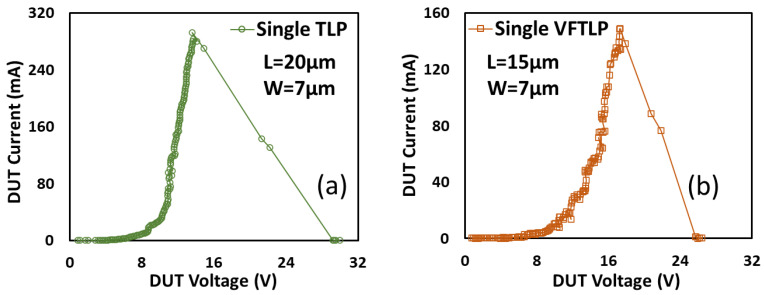
Measurement of single-crystalline gNEMS samples showing robust ESD current-handling capability (I_t2_): (**a**) TLP zapping, and (**b**) VFTLP zapping [44].

**Figure 24:**
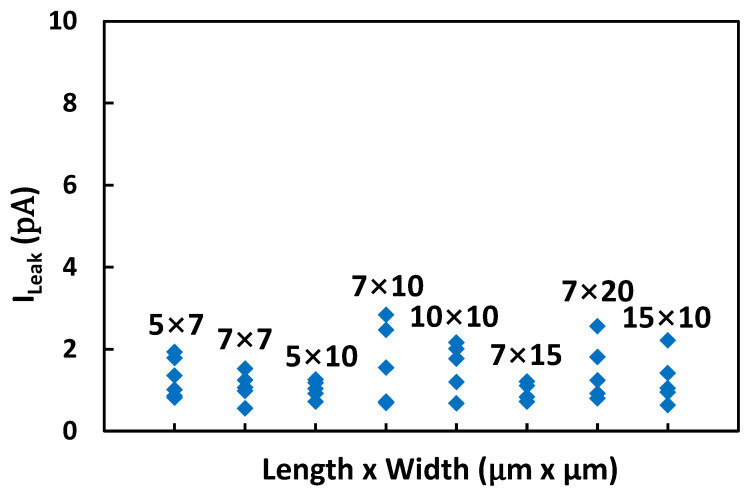
Measurement statistics showing extremely low leakage currents I_leak_ for gNEMS devices under TLP zapping [44].

The first nontraditional ESD-protection device was a two-terminal (2T) graphene-based NEMS switch (gNEMS) device, as depicted in Figure 17. Unlike traditional in-Si PN-based active electronic device ESD protection [43], gNEMS is a mechanical switch comprising a suspended graphene membrane over a cavity with its two electrodes, i.e., graphene ribbon as the anode (A) and the Si substrate as the cathode (K), connected to an I/O pad and ground (GND or V_SS_) and/or supply pad (V_DD_). During normal IC operations, gNEMS remains OFF, hence not interfering with IC functions. During an ESD event, the fast ESD transient generates a strong electrostatic force that pulls the suspended graphene membrane downward to the conducting bottom electrode. When the graphene ribbon touches the K terminal, gNEMS turns ON to form a low-R conduction channel to discharge the incident ESD pulse, hence protecting the IC. Uniquely, gNEMS is a cavity-based mechanical device, thus theoretically introducing negligible C_ESD_, I_leak_, and noises during normal IC operations. In addition, the gNEMS ESD switch is made in the CMOS BEOL deck, above the Si substrate (i.e., above-IC), which ideally does not consume Si area and will make chip-layout planning much easier. The gNEMS ESD switch offers a new ESD-protection mechanism and ESD-protection device structure. The new gNEMS ESD switch concept was first validated experimentally using polycrystalline graphene grown by the CVD method and a CMOS-compatible device-fabrication process flow [43]. As depicted in Figure 18, fabrication of a gNEMS switch device starts with a phosphorus-doped silicon wafer (a), followed by growing a SiO_2_ layer of 250 nm thick by thermal oxidation (b), then a Si_3_N_4_ layer of 100 nm is deposited by plasma-enhanced chemical vapor deposition (PECVD), followed by etching an opening using reactive ion etch (RIE) (c), then a CVD-grown graphene film is transferred to the Si substrate over the opening in the Si_3_N_4_ layer followed by graphene patterning by RIE etching (d), next, Pd (10 nm) and Au (90 nm) electrodes are created by e-beam deposition and lifting off (e), and finally, HF vapor is applied to etch the SiO_2_ underneath the opening to release the suspended graphene membrane to form a gNEMS device (f), as shown in Figure 17 (Inset). Comprehensive ESD measurements were conducted by DC sweeping and TLP and VFTLP ESD zapping tests for a large set of gNEMS prototypes. Figure 19 shows expected gNEMS switch turn-on by simple DC sweeping test and desired dual-directional transient ESD discharging I-V characteristics by TLP zapping, readily validating the new gNEMS ESD switch concept. Figure 20 depicts the TLP-measured ESD triggering voltage V_t1_ for gNEMS devices of various dimensions, showing a wide range of adjustable V_t1_, desirable for practical ESD-protection designs. The gNEMS devices were further improved by using single-crystalline graphene films grown using an improved CVD method, which shows much improved ESD switching and reliability performance [44]. Figure 21a depicts the Raman spectrum for polycrystalline and single-crystalline graphene films, confirming their crystalline structures. ESD performance comparison for polycrystal and single-crystal gNEMS device samples is presented in Figure 21b,c under both DC sweeping test and TLP zapping, which readily confirms that the single-crystal gNEMS device outperforms its polycrystal counterpart, attributed to the outstanding material properties of the single-crystal graphene membrane. To evaluate the design reliability (durability) of gNEMS devices, 110-fold repeat TLP and VFTLP zapping tests were conducted for samples, and the measured I-V characteristics remained very stable, as shown in Figure 22, confirming the superior quality of the single-crystal gNEMS devices fabricated. Upper-limit TLP and VFTLP zapping tests were also performed for single-crystal gNEMS samples to explore their ESD current-handling capability, as shown in Figure 23. These revealed that the single-crystal gNEMS devices had outstanding ESD robustness, achieving a record maximum ESD current density of J_t2_~1.19 × 10^10^ A/cm^2^ under TLP testing and J_t2_~6.09 × 10^9^ A/cm^2^ under VFTLP stressing. This is equivalent to a record HBM ESD capability of ~178 KV/µm^2^, compared over ~7.5 V/µm^2^ for a typical in-Si SCR ESD-protection device, which is generally considered the most robust in-Si ESD-protection device. VFTLP testing confirmed that the new gNEMS device featured ultrafast switching, at least ~100 ps, largely attributed to the mechanical properties and superhigh Young’s modulus of graphene film. Figure 24 shows measured leakage currents for gNEMS samples, showing negligible leakage of ~1′s pA, highly desirable for advanced ICs and superior to its in-Si PN-type ESD-device counterparts. Overall, the new above-IC graphene-based gNEMS ESD-protection structure has the potential to overcome the ESD design overhead problem inherent to any traditional in-Si PN-based ESD-protection structure. Fundamentally, the motivation for using graphene for ESD protection is to leverage the unique material properties of graphene, e.g., ultrahigh carrier mobility, superior thermal conductivity, outstanding mechanical strength, and super Young’s modulus [45,46,47]. It is noteworthy that other possible 2D materials of similar properties may also be explored for making novel ESD-protection structures.

## 5. In-BEOL Nano-Crossbar Array ESD-Protection Structure

**Figure 25:**
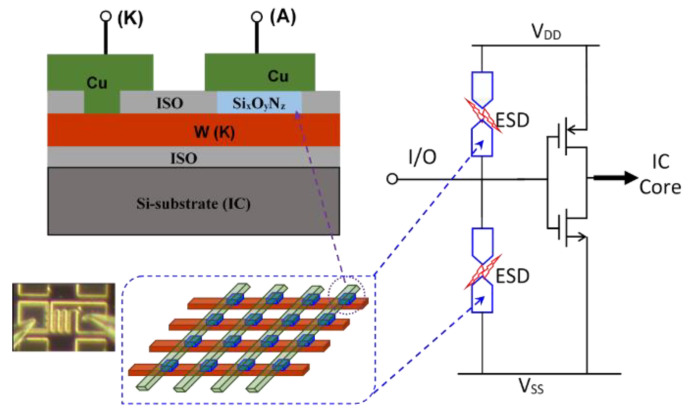
Concept of above-IC nano-crossbar array ESD protection.

**Figure 26:**
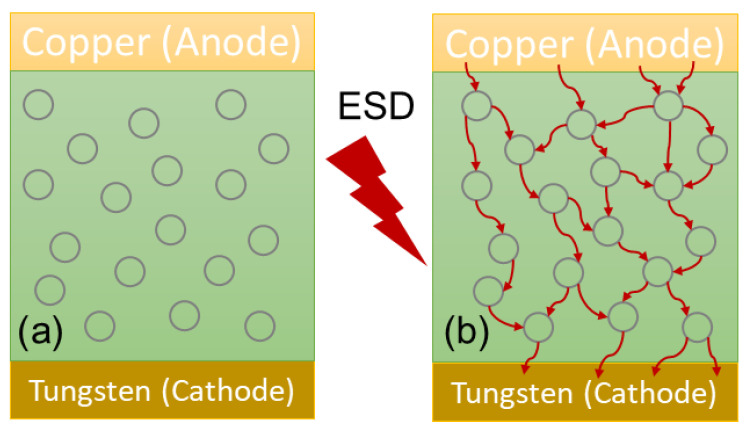
A dispersed local ESD tunneling model was proposed to explain the ultrafast ESD discharge characteristics of a new nano-crossbar array ESD-protection structure: (**a**) Cu ions pre-diffusion and (**b**) local tunneling [48].

**Figure 27:**
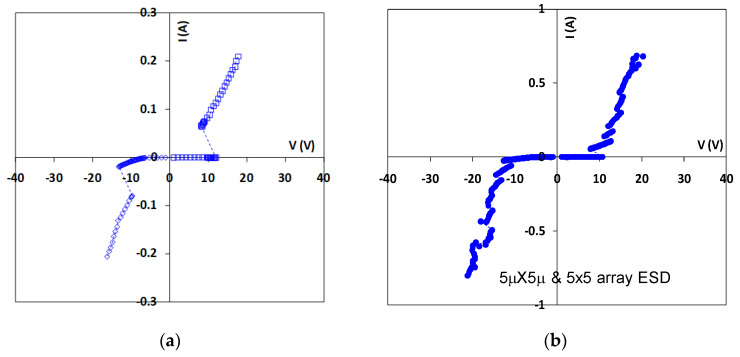
TLP testing of sample nano-crossbar ESD-protection structures shows dual-directional ESD discharge I-V characteristics: (**a**) single-node 5 μm × 5 μm ESD device, and (**b**) 5 × 5 nano-crossbar array ESD device [48].

**Figure 28:**
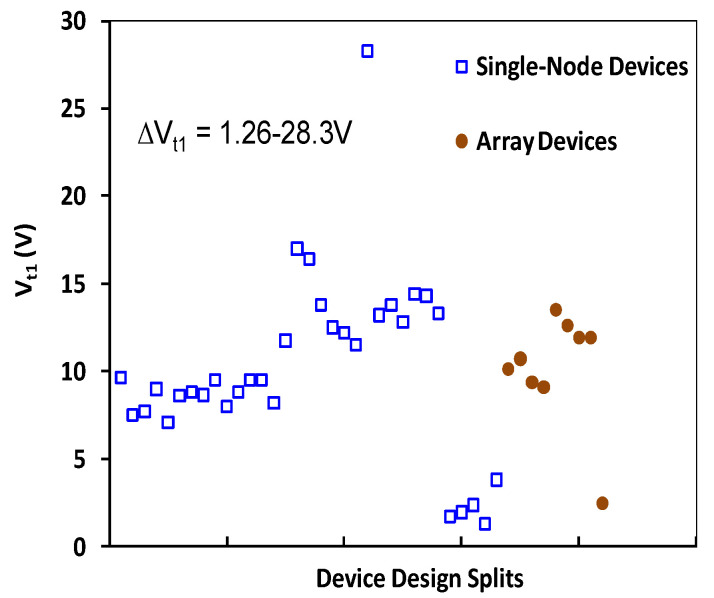
Measured ESD triggering voltages for sample ESD devices show V_t1_ tunability by design [49].

**Figure 29:**
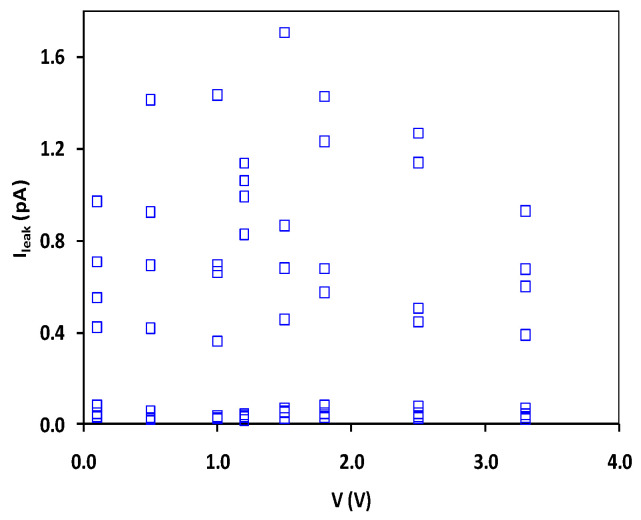
Measured leakage currents for nano-crossbar ESD devices show extremely low ESD-induced I_leak_ [49].

To address the ESD design overhead problem inherent to traditional in-Si PN-type ESD protection structures, a novel in-BEOL phase-changing type nano-crossbar ESD-protection concept has also been proposed and validated experimentally [48,49]. Similarly, the uniqueness is that this ESD device relies on a phase-changing phenomenon for ESD discharging, not any active PN-type devices, and can be made above-IC (in CMOS BEOL), instead of residing inside-Si. Another key advantage of the new nano-crossbar ESD device is with its dual-directional ESD discharging features, which serve to dramatically reduce the total number of ESD devices needed on a chip for constructing a whole-chip ESD-protection network. The new nano-crossbar ESD-protection device structure is depicted in Figure 25, where each crossbar node is a 2T device containing two electrodes (A and K) sandwiched between a phase-changing insulator. The A and K electrodes are connected to bonding pads on a chip. During normal IC operations, a nano-crossbar ESD device stays OFF due to the insulator separating A and K terminals, hence not interfering with IC functions. During an ESD event, the strong transient electrical field induced by an incoming ESD pulse will trigger phase changing in the insulator and turn ON the device to form a low-R ESD conduction path to discharge the ESD pulse into GND, hence providing ESD protection. After the ESD surge is over, the nano-crossbar ESD device will return from ON to OFF state and normal IC operation will resume. The small scale of a nano-crossbar node device seems to be important to retaining the nanoscale phase-changing behavior to ensure ultrafast switching, as confirmed in VFTLP testing, which is explained by a new dispersed local ESD tunneling model depicted in Figure 26. The nano-crossbar ESD design function mechanism follows: annealing drives Cu ions into the insulator materials, under ESD stressing, free carriers will hop over the Cu ion islands through a local tunneling effect, hence realizing ultrafast phase-changing conduction via the local tunneling effect (not a filament conduction mechanism). To ensure high ESD current-handling capability, a nano-crossbar array ESD-protection structure will be used in practical designs to ensure high ESD robustness. The nano-crossbar array ESD-device prototypes were fabricated using a CMOS-compatible process and were characterized by comprehensive ESD zapping testing. Figure 27 depicts the TLP-measured ESD I-V characteristics that readily show the expected dual-directional ESD discharge curve. Furthermore, multiple ESD triggering points appear in the ESD discharge I-V curve for an array ESD structure, attributed to nonsimultaneous phase-changing actions of all individual nano-crossbar nodes within an array. By careful device design (e.g., device dimensions, insulator materials, etc.), the critical ESD triggering V_t1_ can be adjusted, from a few to a few tens of volts in the report, which is very useful in practical ESD-protection designs, as shown in Figure 28. TLP testing reveals very high ESD-protection capability for the new nano-crossbar array ESD-protection structures, e.g., I_t2_~8.11A for a 5 × 5 array device [48]. VFTLP zapping confirms that the new nano-crossbar ESD device can respond to an ultrafast ESD pulse of ~100 ps. Measurement also shows that ESD-induced leakage is extremely low, i.e., <2 pA, as depicted in Figure 29, due to using an insulator medium between the A and K electrodes. Overall, the work clearly shows the functions and potential of the new in-BEOL nano-crossbar array ESD protection for future chips, overcoming the ESD design overhead problem inherent to traditional in-Si PN-type ESD protection structures.

## 6. Graphene Interconnects for on-Chip ESD-Protection Circuits

**Figure 30:**
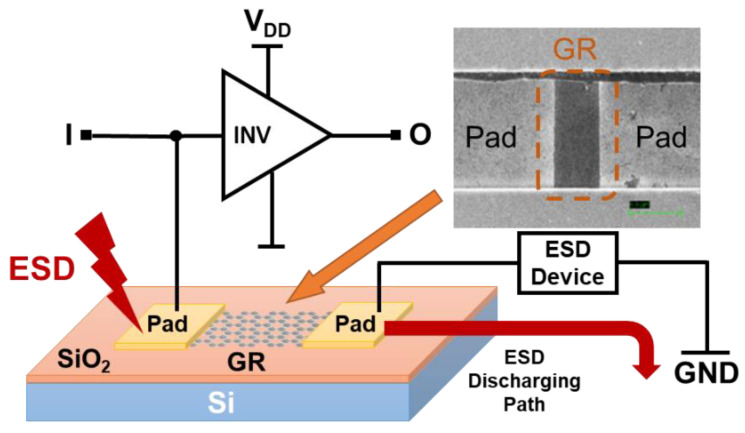
Concept for graphene GNR interconnects for on-chip ESD protection circuits. Inset: photo for a graphene nanoribbon wire fabricated.

**Figure 31:**
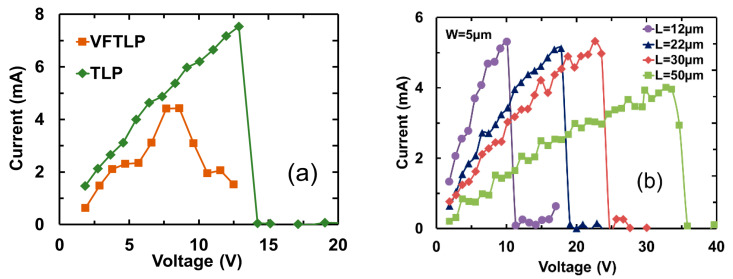
Measured transient ESD I-V characteristics for graphene nanoribbon samples: (**a**) TLP versus VFTLP zapping, and (**b**) TLP I-V curves for GNR wires of different lengths showing impacts on V_C_ [50].

**Figure 32:**
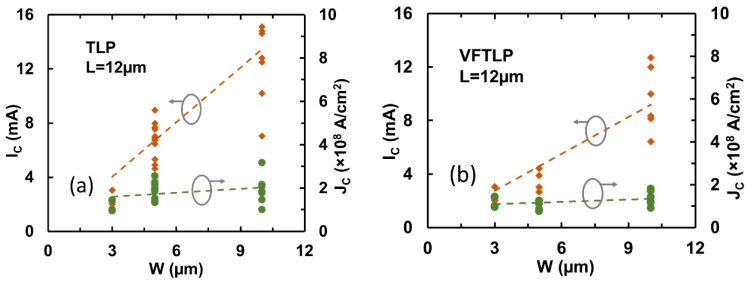
Statistics for I_C_ and J_C_ for GNR wire samples show effect of width variation, W = 3 µm, 5 µm and 10 µm at a fixed L = 12 µm: (**a**) by TLP zapping, and (**b**) by VFTLP zapping [50].

**Figure 33:**
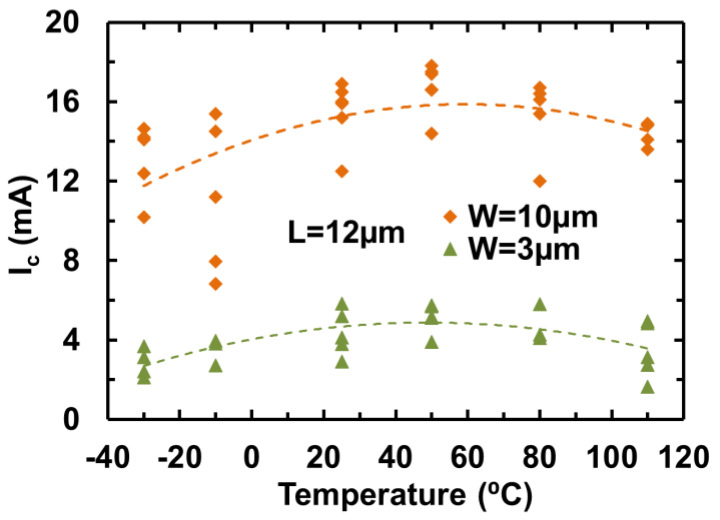
Statistics for TLP-measured I_C_~T behavior (−30 °C to +110 °C) for GNR wire samples show optimal annealing temperature [50].

Similar to any complex ICs, metal interconnects are becoming a design challenge for advanced chips, due to the inevitable parasitic effects associated with the massive metal interconnects, such as capacitive coupling and IR drop effects. Interconnects can be an even bigger design challenge for on-chip ESD-protection circuits, because the large ESD current pulses can readily damage metal interconnects that are normally minimized in IC designs to reduce the parasitic effects. To address this ESD design challenge, graphene nanoribbon (GNR) was studied as a potential solution for ESD interconnects on a chip [47,48]. The motivation was obviously with the unique electrical, thermal, and mechanical properties of graphene materials, i.e., ultrahigh mobility, superior thermal conductivity, and outstanding mechanical strength [45,46,47], all of which are desirable features for ESD protection. In the experiments, a large sample set of GNR wires of varying dimensions (length L, width W, and layer number) were designed using CVD-grown graphene films in both polycrystal and single-crystal structures [50,51]. Figure 30 depicts the application scheme of using GNR wires for on-chip ESD protection. Figure 31 presents measured ESD discharge I-V characteristics for GNR wire samples using TLP and VFTLP zapping (a) and for samples with varying L (b), which readily shows the critical voltage (V_C_) and current (I_C_) of GNR wires measured. Obviously, I_C_ increases as L becomes longer, due to series resistance that increases heating. The influence of GNR width (W) on I_C_ is given in Figure 32, which clearly shows that I_C_ increases for wider GNR wire, due to reduced R. The thermal breakdown current density (J_t2_) appears to be insensitive to W, as expected. The effect of annealing temperature on GNR wires is depicted in Figure 33, revealing a somewhat optimal temperature for graphene treatment, i.e., T_opt_~50–60 °C for I_t2-opt_, suggesting optimization in GNR fabrication to ensure better ESD robustness. It has also been reported that using single-crystal graphene GNR wires for ESD protection achieved much improved ESD robustness over using polycrystal GNRs, due to optimization of graphene materials [51]. In principle, if GNR wires are used to replace traditional Al/Cu metal interconnects for on-chip ESD protection circuits, one can either dramatically narrow the wire width to reduce ESD metal-induced parasitic effects or achieve a much higher ESD-protection level using the same ESD wire width. Future research should study the failure mechanisms and durability of GNR wires by repeated ESD zapping tests.

## 7. Conclusions

In summary, there seems to exist a consensus that heterogeneous integration opens a door to make future CMOS chips smarter in the post-Moore era, because HI technologies can bring in many and various heterogeneities to system chips at material, device, fabrication, circuit, and architecture levels. For years, research efforts have led to many advances in HI technologies, making it impractical to provide a single-paper review of everything in the field. This article provides a topical overview of a few key advances in HI technologies and structures that have been validated in CMOS platforms experimentally. The novel HI structures reviewed in this paper include stacked-via magnetic-core inductors, in-BEOL metal wall structure for global flying crosstalk isolation, above-IC graphene gNEMS switch and nano-crossbar array ESD-protection structures, and using graphene nanoribbons to replace Cu/Al for ESD interconnects. These examples strongly support the vision that HI technologies can be a viable solution for smart future chips, predominant still in CMOS platforms. It is noteworthy that while heterogeneous integration technologies can enrich heterogeneities at all levels in a microsystem, Si CMOSs will remain the foundation upon which function diversification will be built, at least in the foreseeable future, for two main reasons: the maturity of Si CMOS IC technologies and the economy of Si materials. Ideally, any non-Si CMOS-based technologies (i.e., materials, devices, functionalities) loosely referred to as “X” technologies can be heterogeneously integrated into a Si CMOS platform to deliver smarter “CMOS + X” future chips, which generally require a new co-design and co-development philosophy holistically across all layers within a system.

## Data Availability

Data sharing is not applicable to this article.
